# Dual contraceptives and associated predictors in HIV positive women: a case–control study

**DOI:** 10.1186/s12978-022-01475-x

**Published:** 2022-07-29

**Authors:** Alemu Ashore, Desta Erkalo, Ravi Prakash

**Affiliations:** 1 School of Public Health, College of Medicine and Health Sciences, Wachemo University, Hossana, Ethiopia; 2 Hossana Health Center, Hossana, Ethiopia; 3 Present Address: Department of Physical Medicine and Rehabilitation, UT Southwestern Medical Center, Dallas, TX USA

**Keywords:** Dual contraceptives, HIV, AIDS, Sexually active women, Antiretroviral therapy

## Abstract

**Background:**

People living with the human immune deficiency virus (PLHIV) are an important group to address HIV prevention. Mostly, 90% of the HIV cases in children are usually through mother-to-child transmission. Dual contraception (barrier condoms i.e., male, and female condoms) are one of the most effective ways to avoid HIV transmission. Thus, the present study was carried out to establish the predictors associated with the use of dual contraceptives in sexually active HIV positive women in Hossana, Southern Ethiopia.

**Methods:**

An institution based unmatched case–control study among randomly selected 312 sexually active HIV positive women was conducted from February 2021 to May 2021. The data were collected through structured questionnaire and anti-retroviral treatment (ART) cards considering the case-to-control ratio of 1:3. The information was coded, entered into Epi-Info7.0 and exported to SPSS 20.0 for further analysis. A *P-value* < *0.25* in bi-variate analysis was further processed for multi-variate analysis and *P-value* < *0.05* was considered statistically significant.

**Results:**

A response rate of 97.2% was recorded. A significant difference was observed towards the use of dual contraceptives in sexually active HIV positive women living in urban *vs* rural areas (AOR = 0.28; 95% CI = 0.09–0.84), having sexual intercourse with a regular partner (AOR = 3.77; 95% CI = 1.48–9.55) and taking first initiation to use (AOR = 0.05; 95% CI = 0.02–0.11).

**Conclusion:**

The determinants associated with lower use of dual contraceptives were residing in rural areas, sexual intercourse with a regular partner and low initiation rate at first time for use of dual contraceptives. Therefore, we strongly recommend that open discussion about sexually transmitted infections like HIV and their prevention, providing adequate facilities in rural areas can help to prevent HIV transmission and reduce the disease burden. The health professionals are encouraged to organize awareness campaigns in rural areas for use of dual contraceptives among PLHIV.

## Plain language summary

HIV/AIDS has become a serious global threat to human beings. People living with HIV are an important group that needs attention. About 90% of the children get HIV infection through their mothers that involved mother-to-child transmission of HIV. Therefore, World Health Organization has focused on the use of condoms to prevent the transmission of sexually transmitted diseases (STDs) and to avoid unwanted pregnancies. Out of these contraceptives, the use of dual contraceptives has been considered the best to prevent the transmission of STDs/HIV. Therefore, the present study tried to identify the use of dual contraceptives (barrier condoms i.e., male, and female condoms) and factors associated with its use in sexually active HIV women in Hossana, SNNPR, Ethiopia. We conducted a case–control study and recruited 312 sexually active HIV positive women that were on antiretroviral therapy and visiting the ART clinics for their follow-ups. The present investigation revealed that women living in rural areas were not aware about the use of dual contraceptives when compared to urban areas. Also, it was observed that women, those involved in sexual activities with multiple partners and had an open discussion with their partners about using dual contraceptives at first were more when compared to others. Although, the use of dual contraceptives among HIV positive women living in Hossana was low. Hence, this needs more focused awareness programs in rural areas and people having sexual intercourse with regular partners.

## Background

Human Immunodeficiency Virus/Acquired Immune Deficiency Syndrome (HIV/AIDS) is a devastating infection and serious threat to mankind. HIV usually transmits through contact with bodily fluids containing virus or infected cells. Most common transmission routes are blood, semen, vaginal fluid, and breast milk (MTCT; mother-to-child transmission). HIV mainly infects CD4 + T cells that lead to their loss in circulation and weakens the immune system [1]. Populations at risk include female sex workers (FSW), men having sex with men (MSM), injection drug users (IDUs), divorcee and widows, HIV negative partners in discordant couples, long distance drivers, migratory workers, laborers in factories, construction workers, workers in massage houses, local drink, and shisha houses, etc. [2].

By the end of 2020, approximately 37.7 million (30.2–45.1 million) people were living with HIV (PLHIV) and 0.68 million died. Out of 37.7 million PLHIV, 36.0 million were adults and 53% of all these PLHIV were women and girls. A total of 6.1 million people observed were not aware that they are living with HIV [3]. Severe effects of the HIV/AIDS epidemic have been observed in low- and middle-income countries. HIV is disproportionately concentrated in sub-Saharan Africa (SSA) and has remained a significant cause of morbidity and mortality [4, 5]. In 2017, in SSA alone 75% people died, and 65% new infections appeared due to HIV. Despite global efforts to reduce HIV burden and rapid rise in the use of anti-retroviral therapy (ART), almost 34% of the people in Eastern & Southern Africa and 60% in Western & Central Africa living with HIV are still not receiving any kind of treatment [6]. In SSA, women infected with HIV and not receiving antiretroviral therapy (ART) have been observed with increased risk of anemia and adverse pregnancy outcomes like preterm birth, low birth weight (LBW), small for gestational age (SGA) and stillbirths [7, 8].

In 2017, total number of HIV infections in Ethiopia was 722,248. The highest estimate of HIV prevalence among adult population was observed in Addis Ababa (5%) followed by Gambella region (4%) [9]. One of the major risk factors for HIV exposure in Gambella is low coverage of male circumcision [10], whereas, population density was another risk factor for high HIV positivity in Addis Ababa [11]. On the contrary, the Southern Nations, Nationalities and Peoples’ Region (SNNPR) have lower prevalence (0.5%) in Ethiopia [12]. These data indicate that in Ethiopia, the HIV prevalence is geographically variable, and women have HIV prevalence twice (1.2%) as compared to men (0.6%) [13].

Antiretroviral therapy (ART) lowers the viral load, but altogether the use of ART and condoms better prevents further transmission of HIV. The use of male and female condoms altogether (dual or barrier contraceptives) has been proved successful in protecting against HIV when compared to other contraceptive methods (hormonal and others). Women using hormonal contraceptives are encouraged to use female condoms to protect themselves against HIV [14]. The likely reasons behind this are its low cost, high effectiveness, avoids unwanted pregnancies and thus, prevents maternal morbidity and mortality [15]. Various factors influence the acceptance or denial of the use of a contraceptive by Ethiopian women such as personal and partner’s choice, availability of methods & services, cultural & religious taboos and possible adverse effects of contraceptives.

At present, several methods of contraception are in use, such as hormonal (pills), vaginal ring, injections, intrauterine contraceptive devices (IUDs) and non-hormonal methods like copper IUDs, condoms, vaginal sponges, etc. Out of these, oral contraceptives, IUDs, and sterilization have been observed effective. But still, these methods are unable to prevent HIV transmission. Despite HIV care and treatment programs and adherence to ART, the use of dual contraceptives has been considered the best approach to avoid vertical transmission and unintended pregnancies.

Various cross-sectional studies have been conducted in the past on a dual contraceptive utilization that determined the prevalence of a rare event in any population. Therefore, we conducted a first case–control study considering the use of dual contraceptives (male and female condoms) as a case and other contraceptive method as controls to identify the relative importance of a predictor variable or associated factors affecting the use of dual contraceptives. Considering factors that might influence the use of dual contraceptives our study will help to devise local interventions by stakeholders and health administration.

Thus, the present study was carried out to establish the predictors associated with the use of dual contraceptives (male and female condoms) in sexually active HIV positive women taking highly active antiretroviral therapy (HAART) in Wachemo University Nigist Eleni Mohammad Memorial Comprehensive Specialized Hospital (WCUNEMMCSH) and Hossana Health Center (HHC), Hossana.

## Methods

### Study design, area and period

An institution based unmatched case–control study was conducted among antiretroviral therapy users in WCUNEMMCSH and HHC, Hossana, Hadiya zone, Southern Nations, Nationalities and Peoples’ Region (SNNPR). The study was conducted from February 2021 to May 2021.

### Source population

The number of people living with HIV recorded and enrolled in ART were 1587. According to institutional data, out of 1587, 867 HIV positive sexually active women were on antiretroviral therapy. The source population consisted of sexually active HIV positive women in the age group of 18–49 years attending chronic HIV/AIDS care clinic.

### Study population

#### Case

Sexually active HIV positive women in the age group of 18–49 years used dual contraceptives (barrier methods, i.e., male and female condoms) during the study period and attended ART follow-up clinics in WCUNEMMCSH and HHC, Hossana**.**

#### Control

Sexually active HIV positive women in the age group of 18–49 years did not use dual contraceptives (used either one barrier condom or any other contraceptive method) during the study period but attended ART follow-up clinics in WCUNEMMCSH and HHC, Hossana.

### Inclusion criteria

#### Case

All sexually active HIV positive women in the age group of 18–49 years used dual contraceptives (barrier methods, i.e., male and female condoms) during the study period and attended ART follow-up clinics in WCUNEMMCSH and HHC, Hossana.

#### Control

All sexually active HIV positive women in the age group of 18–49 years did not use dual contraceptives (used either one barrier condom or any other contraceptive method) during the study period but attended ART follow-up clinics in WCUNEMMCSH and HHC, Hossana.

### Exclusion criteria

Individuals unable to communicate verbally/seriously ill at the time of data collection.

### Sample size determination and sampling techniques

#### Sample size determination

The sample size was calculated using Epi info 7.0 considering control to case ratio of 3:1, expected percentage of controls exposed 31%, expected percentage of cases exposed 50.2%, 95% level of confidence, 80% of the power and 10% of non-responders [16]. Finally, a total sample size of 321 individuals (80 cases and 241 controls) was calculated. The calculated sample size was in line with reference to a previous study of determinants of dual contraceptive utilization among HIV positive women [17].

#### Sampling procedures/techniques

**Fig. 1:**
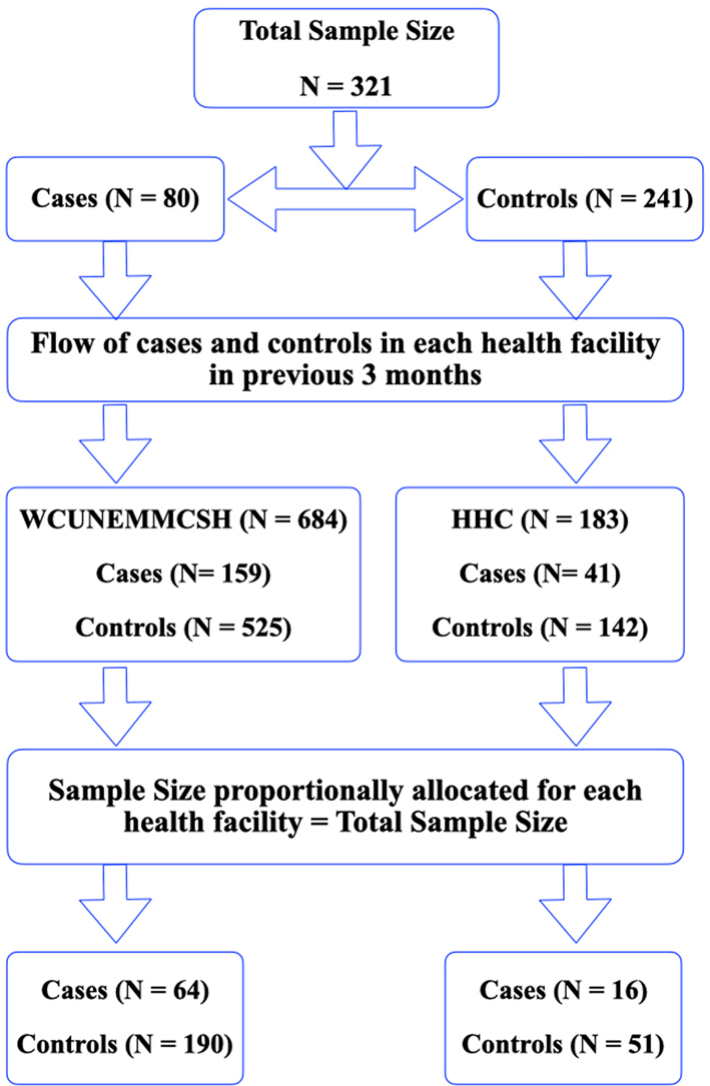
Schematic representation of sample size distribution in WCUNEMMCSH and HHC

Hossana town has two public health facilities: i) Wachemo University Nigist Eleni Mohammed Memorial Comprehensive Specialized Hospital and ii) Hossana Health Centre. The sampling frame for cases and controls from selected public health center or hospital was obtained from ART registration records. The sample size was proportionally distributed to each health facility based on Health Management Information System (HMIS) and reported existing case and control flow in the previous three months (Fig. 1). Considering pre-specified sample size in each arm (cases and controls), we selected the cases purposefully from ART registration records and controls were selected using random sampling techniques until the required sample size was achieved.

### Study variables

#### Dependent variable

##### Dual contraceptive utilization

This refers to the sexually active HIV positive women who used two contraceptives simultaneously [i.e.., barrier method (male and female condom)] used in every sexual encounter in the last six months preceding the study.

#### Independent variables

##### Socio-demographic characteristics

These include age, educational status, marital status, place of residence, ethnicity, religion, occupation, and wealth index.

##### Service-related variables

These include counselling about contraceptive use, accessibility of family planning services, satisfaction and duration of ART, discussion with their partners, CD4 count, time of HIV diagnosed, side effects, HAART users & knowledge of contraceptive use as well as knowledge scores.

##### Social and cultural variables

These variables include religious influence, peer support and support of the partner.

##### Reproductive health variables

These include parity, gravidity, number of live children, discussion with husband, discussion with health care providers, family planning counselling, pregnancy initiation and experience of contraceptives.

##### Risky sexual behaviour variables

These include sexual intercourse in past 6 months without using condom and having multiple sexual partners.

### Data collection procedures and quality control

A structured questionnaire [17–19] was used to collect data and modified according to local study settings. The questionnaire consisted of sociodemographic and economic characteristics (age, women’s and husbands’ education level, marital status, wealth index and place of residence), reproductive variables (parity, gravidity, number of live children, discussion with husband, discussion with health care providers, family planning counselling, intention for pregnancy, knowledge of contraceptives), risky sexual behavior patterns (sexual intercourse in the past six months without condom usage and having multiple sexual partners) and factors related to HIV and STIs (clinical WHO staging, disclosure of HIV status, CD4 count, time of HIV diagnosed and HAART user). The data were collected through face-to-face interviews using structured and pre-tested questionnaire. The questionnaire was initially prepared in English and translated to Amharic and local language i.e., Hadiyissa and back to English to ensure consistency of the content. Five nurses and one public health officer (PHO) were recruited as data collectors and supervisor, respectively. Two day’s training was provided to data collectors towards interviewing techniques and ethical concerns. A pre-test was conducted on questionnaire on 5% of the total sample size in similar settings (Gimbichu district hospital). An alpha value of 0.67 was observed and minor modifications were done in the questionnaire. The data collection was closely supervised by a supervisor and principal investigator daily.

### Operational definitions

#### Cases

Sexually active HIV positive women in the age group of 18–49 years used dual contraceptives (barrier methods, i.e., male and female condoms) for ≥ 6 months and attended ART follow-up clinics.

#### Controls

Sexually active HIV positive women in the age group of 18–49 did not use dual contraceptives (barrier methods, i.e., male and female condoms) in past 6 months but attended ART follow up clinics.

#### Accessibility of family planning services

This refers to the distance from patients’ residence to the health institution (i.e., 5 km or ≤ 30 min walking time was considered as accessible) [20].

#### Knowledge of contraceptive use

This belongs to a total of five dichotomized questions; 1 an incorrect answer and 2 for correct answer about contraceptive use. Women who answered and scored median or above were considered to have good knowledge and below median score were considered to have poor knowledge [21].

### Data processing and analysis

The data were entered, sorted, edited, and cleaned for missed values. The data were analyzed using SPSS version 20.0 for further analysis. The correlation between dependent and independent variables was primarily analyzed by bivariate analysis to find any significant association. A *P-value* < *0.25* observed during bivariate analysis were further processed for multivariate analysis to check the probable effect of confounders. A *P-value* < *0.05* was considered statistically significant.

### Ethical consideration

The ethical clearance was obtained from an ethical review committee of College of Medicine and Health Sciences. Written permissions were obtained from higher authorities of WCUNEMMCSH and Hossana Health Center. A verbal and written informed consent for participation was obtained from each study participant. Before enrolling any of the eligible study participants, the purpose, benefits, and confidential nature of the study was described. The discussions between the data collectors and the respondents solely took place in a separate room. The confidentiality of study individuals was maintained, and all the data were de-identified before analysis.

## Results

### Socio-demographic characteristics

A response rate of 97.2% was observed. Nine individuals were excluded due to incomplete data or no response. Finally, a total of 312 individuals [25% cases (N = 78) and 75% controls (N = 234)] were analyzed. The majority of the cases and controls were in the age group of 30–39 years [(48.7% cases (N = 38) and 55.1% controls (N = 129)], belonged to Hadiya ethnicity [(62.8% cases (N = 49) and 69.2% controls (N = 162)] and were protestants [(70.5% cases (N = 55) and 64.1% controls (N = 150)].

Further stratification of data into the educational background revealed women attended at least secondary school were in the majority for cases 29.5% (N = 24) and women who attended at least primary school were in the majority of the controls 38% (N = 89). The housewives consisted of 50% (N = 39) cases & 70.9% (N = 166) controls, whereas, urban residents contained the majority of the cases 91% (N = 71) & controls 83% (N = 188). Among substance users it was observed that spouses of 24.4% (N = 19) cases and 11.5% (N = 27) controls were chewing khat (a stimulant which is used to cause excitement, loss of appetite and euphoria in Ethiopia). Further wealth index revealed that HIV positive women with poor wealth consisted of a number of cases (N = 20; 25.6%), whereas, medium income status consisted of maximum number of controls (N = 62; 26.5%) (Table 1).

**Table 1:** Socio-demographic characteristics of participants in determinants of dual contraceptive utilization among HIV positive women

Variables	Categories	Cases N = 78 (%)	Controls N = 234 (%)	P-value
Age (in years)	18 to 29	33 (42.3)	73 (31.2)	0.125
	30 to 39	38 (48.7)	129 (55.1)	
	40 to 49	7 (9)	32 (13.7)	
Ethnicity	Hadiya	49 (62.8)	162 (69.2)	0.102
	Gurage	6 (7.7)	17 (7.3)	
	Amharic	10 (12.8)	10 (4.3)	
	Kembata	9 (11.5)	21 (9)	
	Silte	3 (3.8)	17 (7.3)	
	Others^*^	1 (1.3)	7 (3)	
Religion	Protestant	55 (70.5)	150 (64.1)	0.035
	Orthodox	19 (24.4)	47 (20.1)	
	Muslim	4 (5.1)	30 (12.8)	
	Catholic	0 (0)	7 (3)	
Educational Status	Can’t read and write	13 (16.7)	49 (19.9)	0.062
	Can read and write(no grade)	10 (12.8)	41 (17.5)	
	Primary school (1–8)	23 (29.5)	89 (38)	
	Secondary school (9–12)	24 (30.8)	41 (17.5)	
	Colleges and Above	8 (10.3)	14 (6)	
Occupational Status	House Wife	39 (50)	166 (70.9)	0.001
	Merchant	12 (15.4)	33 (14.1)	
	Government Employee	15 (19.2)	14 (6)	
	Daily Laborer	6 (7.7)	15 (6.4)	
	Others^**^	6 (7.7)	6 (2.6)	
Residence	Urban	71 (91)	188 (83)	0.030
	Rural	7 (9)	46 (17)	
Husband use to substance (N = 87)	Alcohols	10 (12.8)	28 (12)	0.041
	Chat	19 (24.4)	27 (11.5)	
	Cigarette	1 (1.3)	2 (0.9)	
Wealth index	Poorest	20 (25.6)	50 (21.4)	0.723
	Poor	20 (25.6)	49 (20.9)	
	Medium	16 (20.5)	62 (26.5)	
	Rich	13 (16.7)	43 (18.4)	
	Richest	9 (11.5)	30 (12.8)	

*corresponds to other ethnicities: Halaba (N=3), Wolaita (N=2) and Tigray (N=3)

**corresponds to non-government employee (N=11)

### Knowledge on dual contraception

We evaluated the knowledge of participants (both cases and controls) about types of contraceptive methods through a structured questionnaire. It was observed that 100% of the cases (N = 78) and 85% (N = 199) controls had prior knowledge about dual contraceptives. A response rate of 96.2% was reported among cases when inquired about contraceptive use during ART, whereas, only 79.8% (N = 186) of the controls had this information. Similarly, a 100% response rate was observed for cases (N = 78) about use of dual contraceptive methods to prevent pregnancy, but, only 85% (N = 199) of the controls reported to have this information. Only 89.7% (N = 70) of the cases had knowledge that sexual intercourse without condom increases the risk for sexually transmitted diseases, whereas, only 58.1% (N = 136) individuals from controls had this prior knowledge. Prior knowledge on the use of most suitable dual contraceptive methods for HIV positive women revealed that 96.2% (N = 76) cases reported use of male condoms. Similarly, 97% (N = 227) participants among controls reported the use of male condoms as a most suitable contraceptive method (Table 2). Further stratification of data about knowledge of dual contraceptives, a high level of knowledge (100%) was observed among cases, whereas, in controls the low level of knowledge (79.9%) was observed (Table 3).

**Table 2:** Knowledge of cases and controls about dual contraception

Variables	Categories	Cases N = 78 (%)	Controls N = 234 (%)	P-value
Have you ever heard about dual contraceptive methods?	Yes	78 (100)	199 (85)	0.0001
	No	0 (0)	35 (15)	
Can contraceptive methods be used while on antiretroviral therapy?	Yes	75 (96.2)	186 (79.8)	0.005
	No	3 (3.8)	47 (20.2)	
Do you know any dual contraceptive utilization that is used in preventing pregnancy?	Yes	78 (100)	199 (85)	0.0001
	No	0 (0)	35 (15)	
Do you have information that sex without condoms poses risk for transmission of STIs?	Yes	70 (89.7)	136 (58.1)	0.0001
	No	8 (10.3)	98 (41.9)	
What is the most suitable dual contraceptive method for HIV positive women?	Male condoms	76 (96.2)	227 (97)	0.845
	Female condoms	2 (3.8)	7 (3)

**Table 3:** Categories of Knowledge level on dual contraception

Levels of knowledge	Range	Cases N = 78 (%)	Controls N = 234 (%)
Low	< 3	0 (0)	187 (79.9%)
Moderate	3	0 (0)	47 (15.1%)
High	> 3	78 (100%)	0 (0)

### Practice on dual contraception

**Fig. 2:**
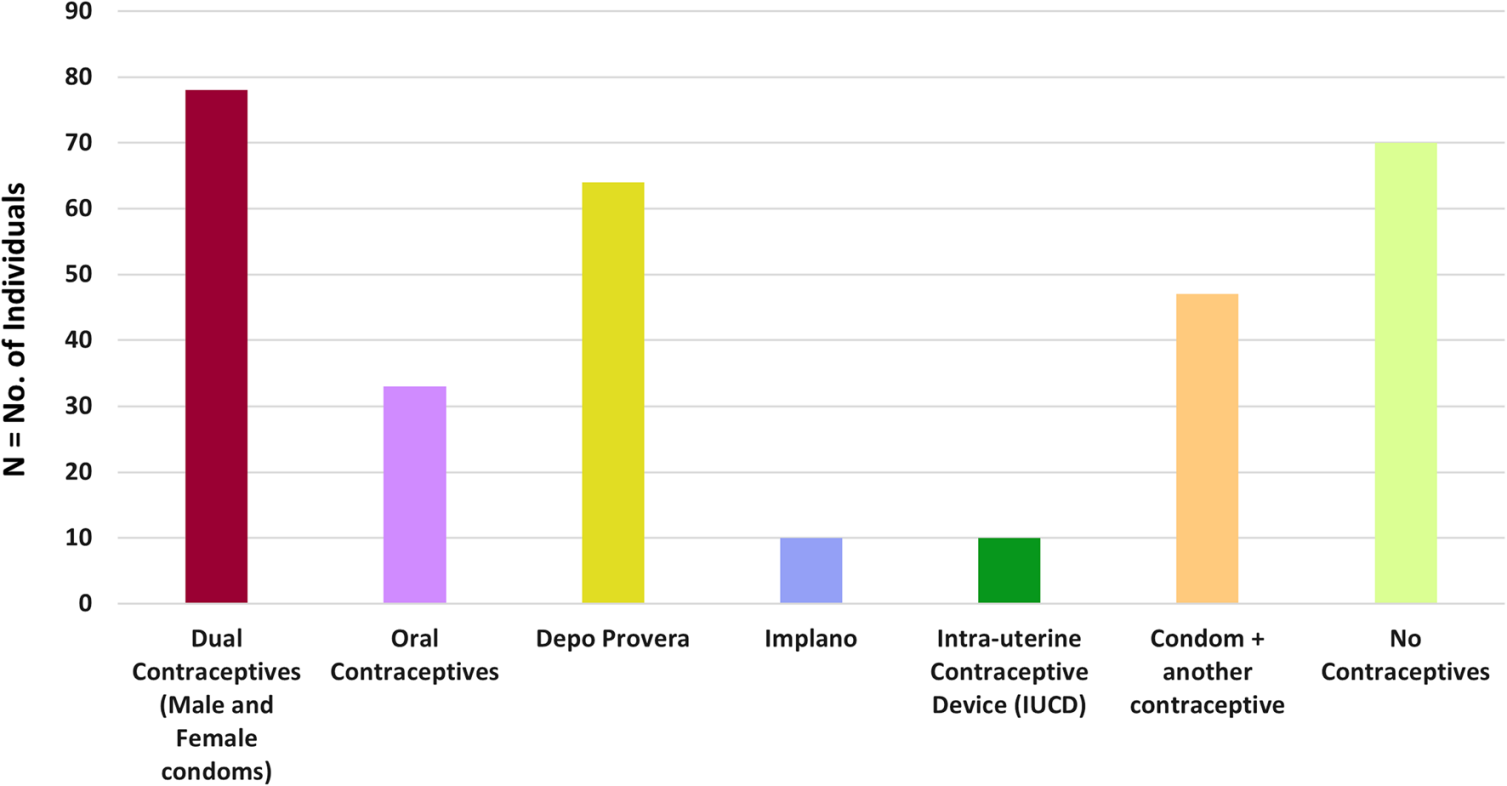
Practice on utilization of dual contraceptives

This was observed that sexually active HIV positive women among cases were using dual contraceptives. Among controls 22.4% (N = 70) did not use any single contraceptive, 10.6% (N = 33) used oral contraceptives, 20.5% (N = 64) were on Depo Provera, 3.2% (N = 10) used implano and 3.2% (N = 10) were using intra-uterine contraceptive device (IUCD) and 15% (N = 47) of participants at the same time used condom plus another contraceptives (hormonal pills, etc.) (Fig. 2).

### Sexual and Reproductive health vs dual contraceptive utilization

A total of 67.9% (N = 53) sexually active HIV positive women from cases and 76.9% (N = 180) from controls married the first time when ≥ 18 years old. About 80.8% (N = 63) of cases and 89.3% (N = 209) of controls reported to have children. It was observed that only 41.2% (N = 28) of cases and 56.9% (N = 115) of controls from HIV positive women had 3–4 children. However, 52.6% (N = 41) of participants from cases expressed their desire to have children in the future, whereas, 59.4% (N = 136) controls denied their intention for more children.

When inquired about the sexual intercourse in past six months, 100% of the cases (N = 78) reported to have had, whereas, only 85.4% (N = 203) controls had intercourse in past six months. Out of 78 cases, 67.9% (N = 53) revealed to have sex with their regular partners, whereas, in controls 90.2% (N = 211) participants were observed for the same. In cases, 100% response rate was observed for the use of condoms. Only, 62.8% (N = 49) of the cases and 59.6% (N = 28) of the controls reported that they preferred condoms to avoid the pregnancy.

Further stratification of data about initiation of condom usage, 71.8% (N = 56) of cases reported using dual contraceptives with mutual decision of the sexual partner, whereas, 77.4% (N = 181) controls did not initiate condom use either herself or due to a mutual decision with sexual partner. It was also reported that 28.2% (N = 22) of the cases and 77.4% (N = 181) controls never discussed about the use of condoms. Only, 74.3% (N = 52) individuals from controls reported reason for not using condoms (preferred to conceive for the pregnancy). One-third of the cases 33.3% (N = 25) and 40.9% (N = 94) controls stayed together with their partners for more than 10–14 years (Table 4).

**Table 4:** Sexual and Reproductive health *vs* dual contraceptive utilization among HIV women attending ART clinic

Variables	Categories	Cases N = 78 (%)	Controls N = 234 (%)	P-value
Age at first marriage	< 18	25 (32.1)	53 (23.1)	0.114
	≥ 18	53 (67.9)	180 (76.9)	
Have child	Yes	63 (80.8)	209 (89.3)	0.051
	No	15 (19.2)	25 (10.7)	
Number of children (N = 269)	1–2	22 (32.4)	42 (20.8)	0.060
	3–4	28 (41.2)	115 (56.9)	
	≥ 5	18 (26.5)	45 (22.3)	
Desire to have a child in the future	Yes	41 (52.6)	95 (40.6)	0.065
	No	37 (47.4)	139(59.4)	
Partners’ desire to have a child in the future	Yes	42 (53.8)	103 (44)	0.132
	No	36 (46.2)	131 (56)	
Sexual intercourse within the last 6 months	Yes	78 (100)	203 (86.8)	0.001
	No	0 (00)	31 (13.2)	
Sex with whom	Regular partner	53 (67.9)	211 (90.2)	0.0001
	Multi-sexual partner	25 (32.1)	23 (9.8)	
Use of condom	Yes	78 (100)	69 (29.5)	0.0001
	No	0 (0)	165 (70.5)	
Do you use condom?	Always	78 (100)	0 (0)	0.0001
	Sometimes	0 (0)	47 (20.1)	
	Never	0 (0)	187 (79.9)	
Reasons for using condom (N = 125)	To prevent pregnancy	49 (62.8)	28 (59.6)	0.178
	My partner is HIV positive	1 (1.3)	1 (2.1)	
	To reduce viral loads	4 (5.1)	9 (19.1)	
	Fear of other STIs	9 (11.5)	3 (6.4)	
	Just health professional’s advice	14 (17.9)	6 (12.8)	
	Others	1 (1.3)	0 (0)	
Initiators of condom use (he/herself or partners’ mutual decision**)**	Yes	56 (71.8)	53 (22.6)	0.0001
	No	22 (28.2)	181 (77.4)	
Reasons for not using contraceptive (N = 70)	I want a child	0 (0)	52 (74.3)	1
	Fear of side effects	0 (0)	4 (5.7)	
	lack of knowledge	0 (0)	7 (10)	
	Other	0 (0)	7 (10)	
Stayed with your partner (N = 305)	≤ 4 years	3 (4)	7 (3)	0.635
	5–9 years	23 (30.7)	69 (30)	
	10–14 years	25 (33.3)	94 (40.9)	
	> 15 years	24 (32)	60 (26)	

### Service-related factors *vs* dual contraceptive utilization

We observed that 87.2% (N = 68) cases and 76.9% (N = 180) controls had ever received different type of counselling in the past three months. Only 25.4% (N = 34) cases and 55.6% (N = 100) controls received counselling towards the use of dual contraceptives. About 71.4% (N = 56) of the cases and 50% (N = 117) controls had a discussion with their partners about using any contraceptive method to delay or avoid pregnancy. Thirty-six (64.3%) of the cases and 87 (76.3%) of controls were involved in joint decision about using contraceptives. When inquired about HIV testing, approximately, 97.4% (N = 76) of the cases and 97.4% (N = 228) controls underwent for HIV test, whereas, 96.1% (N-73) of the individuals from cases and 97.8% (N = 223) controls resulted positive for one of the partners.

Nearly, 78.4% (N = 61) cases and 57.7% (N = 135) controls observed to have CD4 count greater than 500 mm/cells. A total of 71.8% (N = 56) cases and 90.6% (N = 212) controls were already on antiretroviral therapy for more than 24 months. The health status of 94.9% (N = 74) cases and 94.4% (N = 221) controls observed improving after the initiation of antiretroviral therapy. It was observed that 59% (N = 46) cases and 58.5% (N = 137) controls had to wait for > 30 min for counselling or access the services after their arrival at the health facility. Almost 67.4% (N = 31) of cases and 20.8% (N = 44) controls among HIV positive women were supported by their husbands. Also, this was observed that 65.4% (N = 51) of cases and 75.6% (N = 177) controls didn’t face any cultural & social taboos to use contraceptives. Approximately, 61.5% (N = 48) cases and 65.4% (N = 153) controls reported to have no social and cultural factor that can affect women to not to use dual contraceptives. A significant proportion, 73.1% (N = 57) of cases and 77.7% (N = 199) controls reported prior information of male condoms, whereas, 66.7% (N = 52) and 66.5% (N = 153) controls reported the main source of information was health education or health professionals. About 78.2% (N = 61) cases and 69.7% (N = 163) control individuals observed to have a positive attitude about the use of dual contraceptives (Table 5).

**Table 5:** Service related factors of participants in determinants of dual contraceptive utilization

Variables	Categories	Cases N = 78 (%)	Controls N = 234 (%)	P-value
Can use to avoid or delay pregnancy?	Yes	78 (100)	212 (90.6)	0.005
	No	0 (0)	22 (9.4)	
Condom plus another contraceptive	Yes	0 (0)	47 (20.1)	0.0001
	No	78 (100)	187 (79.9)	
Do you use condom plus another contraceptives? (N = 47)	Pills	0 (0)	18 (38.3)	1
	Injectable	0 (0)	22 (46.8)	
	IUD	0 (0)	7 (14.9)	
Which type of dual contraceptive currently use? (N = 242)	Pills + Condom	0 (00)	18 (7.7)	0.0001
	Injectable + Condom	0 (00)	22 (9.4)	
	IUCD + condom	0 (00)	7 (3)	
	condoms only (male and female condoms)	78 (100)	0 (00)	
	Pills	0 (00)	33 ( 14.1)	
	Injectable	0 (00)	64 (27.4)	
	Implanon	0 (00)	10 ( 4.3)	
	IUCD	0 (00)	10 ( 3.8)	
Have you ever received any follow up counseling in the last 3 months?	Yes	68 (87.2)	180 (76.9)	0.052
	No	10 (12.8)	54 (23.1)	
Which type of counselling did you receive? (N = 248)	About condom use	23 (33.8)	39 (21.7)	0.121
	About use of dual contraceptives	34 (25.4)	100 (55.6)	
	About nutrition	11 (16.2)	41 (22.8)	
Have you ever discussed with your partner about using any contraceptive method to delay or avoid pregnancy use?	Yes	56 (71.4)	117 (50)	0.001
	No	22 (28.2)	117 (50)	
Who would be decision maker? (N = 170)	My decision	7 (12.5)	10 (8.8)	0.236
	My partners’ decision	12 (21.4)	17 (14.9)	
	Joint decision	36 (64.3)	87 (76.3)	
	Others	1 (1.8)	0 (0)	
Did your husband or partner get HIV tested?	Yes	76 (97.4)	228 (97.4)	1
	No	2 (2.6)	6 (2.6)	
If yes, What was his result? (N = 304)	Positive	73 (96.1)	22 3(97.8)	0.408
	Negative	3 (3.9)	5 (2.2)	
How much is your recent CD4 count?	< 250 cells/dl	0 (00)	1 (0.4)	0.001
	250-350cells/dl	0 (00)	34 (14.5)	
	350-500cells/dl	17 (21)	64 (27.4)	
	> 500cells/dl	61 (78.4)	135 (57.7)	
Have you started ART?	Yes	78 (100)	234 (100)	1
	12–24 months	22 (28.2)	22 (9.4)	0.0001
	> 24 months	56 (71.8)	212 (90.6)	
How is your health status after ART started?	Improved	74 (94.9)	221 (94.4)	0.708
	Same	4 (5.1)	11 (4.7)	
	Worsened	0 (0)	2 (0.9)	
Time taken for counselling	≤ 30 min	32 (41)	97 (41.5)	
	> 30 min	46 (59)	137 (58.5)	
Have you ever faced the absence of services after reaching the health facility?	Yes	28 (35.9)	67 (28.6)	0.227
	No	50 (64.1)	167 (71.4)	
Who supported you? (N = 117)	Husband	31 (67.4)	44 (58.7)	0.541
	Friends	2 (4.3)	7 (9.9)	
	Relatives	0 (00)	0 (00)	
	Health Professional	13 (28.3)	20 (28.2)	
Is there any cultural practice in your community that prevents you from using contraceptives?	Yes	27 (34.6)	57 (24.4)	0.077
	No	51 (65.4)	177 (75.6)	
Do your spouse believe in any cultural or religious reason to not to use dual contraceptives?	Yes	30 (38.5)	81 (34.6)	0.539
	No	48 (61.5)	153 (65.4)	
Do you have any social stigma?	Yes	19 (24.4)	50 (21.4)	0.581
	No	59 (75.6)	184 (78.6)	
Did you use substance?	Alcohols	8 (10.3)	15 (6.4)	0.155
	Chat	9 (11.5)	15 (6.4)	
	Never	61 (78.2)	226 (87.2)	
What kind of dual contraceptive utilization have you heard before?	Male condom	57 (73.1)	199 (77.7)	0.003
	Female condom	1 (1.3)	7 (3)	
	Male condom + Female condom	20 (25.6)	24 (10.4)	
Where did you get the knowledge or information about contraception?	TV/Radio Newspaper/Internet (Media)	6 (7.7)	12 (5.2)	0.789
	Health Education/ School Health	20 (25.6)	64 (27.8)	
	Doctors/ Nurses (Medical Professionals)	52 (66.7)	153 (66.5)	
	Friends/Family Member/Neighbors	0 (0)	1 (0.3)	
Attitude	Negative Attitude	17 (21.8)	71 (30.3)	0.146
	Positive Attitude	61 (78.2)	163 (69.7)	

### Determinant factors for dual contraceptive utilization among HIV positive women

A significant association was observed between sexually active HIV positive women living in urban *vs* rural areas (AOR = 0.284; 95% CI = 0.096–0.843; P-value = 0.030), had sexual intercourse with regular *vs* multiple sexual partners (AOR = 3.770; 95% CI = 1.487–9.558; P-value = 0.0001) and initiators for usage of dual contraceptives (AOR = 0.050; 95% CI = 0.022–0.113; P-value = 0.0001) (Table 6).

**Table 6:** Multivariate binary logistic regression analysis for dual contraceptive utilization among HIV positive women

Variables	Categories	Cases N = 78 (%)	Controls N = 234 (%)	COR (95%CI)	AOR (95%CI)	P-value
Residence	Urban	71 (91)	188 (83)	1	1	0.030
	Rural	7 (9)	46 (17)	0.403 (0.174–0.934)**	0.284 (0.096–0.843)*	
Sex with whom	Regular partner	53 (67.9)	211 (90.2)	1	1	0.0001
	Multi-sexual partner	25 (32.1)	23 (9.8)	4.327 (2.279–8.218)**	3.770 (1.487–9.558)*	
Type of counselling received (N = 247)	About condom use	23 (33.8)	39 (21.7)	2.198 (0.947–5.100)	5.973 (2.118–16.843)	0.121
	About use of dual contraceptives	34 (25.4)	100 (55.6)	1	1	
	About nutrition	11 (16.2)	41 (22.8)	1.267 (0.586–2.740)	3.113 (1.249–7.757)	
Initiators of dual contraceptive use	Yes	56 (71.8)	53 (22.6)	8.693 (4.865–15.533)**	0.050 (0.022–0.113)*	0.0001
	No	22 (28.2)	181 (77.4)	1	1	

Where 1 = Reference; '*' corresponds to significant difference (*P* < 0.05)

## Discussion

The present study revealed that sexually active HIV positive women living in urban areas, involved in sexual intercourse with regular partners and prior discussion with respective sexual partners about initiation of use of contraceptives showed significant difference.

In low- and middle-income countries residential location plays an important role in determining the outcome of health infrastructure or interventions. The present study showed that about 71.6% HIV positive women living in rural areas were less likely to use dual contraceptives when compared to their urban counterparts. These findings are similar in line to earlier studies in Ethiopia [22, 23]. On the contrary, another study from Northwest Ethiopia reported that urban dwellers use less modern contraceptives when compared to their rural counterparts [24]. One of the likely reasons might be less awareness among HIV positive women in rural areas. Second, in awe of poor health infrastructure, information technology and social taboos in rural areas, women might not be able for a free discussion with their sexual partner to utilize family planning services and dual contraceptives. Other factors might include less awareness about mother to child transmission of HIV and poor physical access to contraceptives in rural areas. On the contrary, women in urban areas have easier access to services and information about dual contraceptives through social media and interpersonal relations.

Our findings observed that HIV positive women who have had sexual intercourse with multi-sexual partners were 3.7 times more likely to use dual contraceptive methods than women having sexual intercourse with their regular partners. Similar observations have been reported from previous studies in Ethiopia and Nigeria [18, 24, 25]. Another study from Michigan observed use of contraceptives four-fold more by young women during their sexual concurrency [26]. Considering the risk of developing an infection people more likely adopt practices to avoid development of infection. Hence, a history of sexual intercourse with multiple sexual partners has significant impact on decision making to use dual contraceptives. Also the single/widowed PLHIV may feel that they are at risk of transmitting the HIV virus to/from their casual sexual partner and might be using dual contraceptives more.

This was observed that HIV positive women who were using dual contraceptives had an open discussion with their sexual partners about contraceptive methods and observed 95% more likely to use than who had no discussion or initiated. Similar observations have been observed from Kenya and in previous studies from Ethiopia [27–30]. The likely reason behind this is that open discussion with their partners always encourages HIV status disclosure, future fertility desires and more importantly helps to manage the life easily. On the contrary, one of the studies from Uganda supports the hypothesis that women who did not disclose their HIV status to their partners or initiated to use dual contraceptives were less likely to use dual contraceptives [31]. The likely reason behind the less use of dual contraceptives might be spouses’ peer faith in religious or cultural taboos to not to use dual contraceptives. The other probable reasons might be different religious thoughts and teachings, different study design, demographic conditions, attitude of the people, ethnic beliefs and the time of contraceptive utilization.

## Strength and limitations of the study

The major strength of the present study is within the small sample size three determinants found associated with lower use of dual contraceptives in control population. The data generated can be used to develop sexual and reproductive educational programs to promote the knowledge and use of dual contraceptives. However, there are a few limitations too. The first and foremost limitation is the sensitivity of the topic. Since, this is a sensitive issue, participants might have been hesitant to answer some of the questions based on their knowledge or experience. Furthermore, the current study was health facility based. Since the present study is a case–control study and earlier studies were cross-sectional, which pose a limitation to compare the findings.

## Conclusion

In this study, urban population, regular sexual partners, and initiation to use dual contraceptive were observed significantly related to the utilization of dual contraceptives. Hence, based on these findings, it is strongly recommended that open discussion about sexually transmitted infections like HIV and their prevention, providing adequate facilities in rural areas can help to prevent further spread of HIV and reduce the disease burden. Health professionals working in ART clinics must organize awareness campaigns and put efforts to increase the number of dual contraceptive users among HIV positive women in WUNEMMCSH and Hossana Health Centers. This needs intervention by the altogether involvement of district health authorities, zonal health authorities, WCUNEMMCSH & other concerned stakeholders. Future research studies should be conducted outside the hospital and health centers to overcome limitations of this study.

## Data Availability

The dataset that supports the conclusions of this study are already included within the article.

## Abbreviations

AIDS: Acquired Immune Deficiency Syndrome
ART: Antiretroviral Therapy
HAART: Highly Active Antiretroviral Therapy
HHC: Hossana Health Center
HIV: Human Immunodeficiency Virus
IDUs: Injection Drug Users
IUDs: Intra Uterine Devices
LBW: Low Birth Weight
MSM: Men having sex with men
MTCT: Mother to child transmission
PLHIV: People Living with HIV
SGA: Small for Gestational Age
SNNPR: Southern Nations, Nationalities and Peoples’ Region
SSA: Sub-Saharan Africa
STIs: Sexually Transmitted Infections
WCUNEMMCSH: Wachemo University Nigist Eleni Mohammad Memorial Comprehensive Specialized Hospital
